# Supplementary material for: Bioinformatics Analysis Highlight Differentially Expressed CCNB1 and PLK1 Genes as Potential Anti-Breast Cancer Drug Targets and Prognostic Markers
Source: Genes (Basel). 2022 Apr 7;13(4):654. doi: 10.3390/genes13040654 (PMC9027215; doi:10.3390/genes13040654)
Supplement: Supplementary file 1 [file genes-13-00654-s001.zip › genes-1649988-supplementary.pdf]

Figure S1. Flow chart of analysis

Figure S2. The demonstration of WGCNA process. **(A)**Sample clustering tree diagram. The y−axis is the height of the cluster tree, and the x−axis represents sample, which finally indicates the clustering of all samples. **(B)** Screening of Soft threshold(power). The y−axis R^2 in the left is the square of the correlation coefficient of log10 (k) and log10 (p (k)), log10 (k) is the logarithm of the node with degree k, log10 (p (k)) is the logarithm of the probability of the node with degree k, and log10 (k) and log10 (p (k)) are negatively correlated. The x-axis is soft threshold (power). The red line is the correlation coefficient R^2 = 0.9. The right is the verification diagram. The y−axis is connectivity and the x−axis is soft threshold (power). **(C)** Clustering tree of DEGs with module colors. The lower part is gene clustering of each module , and the upper part is the corresponding clustering tree.

Figure S3. Immunohistochemical staining data of hub genes in breast cancer tissue samples and normal tissue samples were obtained from the HPA database. The immunohistochemical staining data of *BUB1*, *BUB1B* and *NDC80* in breast tissue were not included in HPA database.

Figure S4. K−M analysis of 13 hub genes except *CCNB1* and *PLK1*. The x−axis is the survival time, and the death time or the last follow−up time of the patient is taken as the final survival time of the patient. The y−axis is survival rate. The samples were divided into "high" (Red) and "low" (Blue) gene expression groups, and the segmentation threshold was the median. $P < 0.05$ was considered statistically significant.

Figure S5. Further analysis of *CCNB1* and *PLK1*. **(A)** The expression level of *CCNB1* in different breast cancer stages. (B) The expression level of *PLK1* in different breast cancer stages. The x−axis is stage, the y−axis is the expression quantity. (C) The expression level of *CCNB1* isoforms. (D) The expression level of *PLK1* isoforms. The C−D x−axis is the different isomers of *CCNB1* and *PLK1*, and the y−axis is the expression level. The thick black or white bar in the middle of each violin chart represents the quartile range, the small black line extending from it represents the 95% confidence interval, and the white dot or black line in the middle is the median.

Figure S6. Expression of *CCNB1* and *PLK1* in other cancer tissues. (A) Expression of *CCNB1* in different cancer tissues and corresponding normal tissues. (B) Expression of *PLK1* in different cancer tissues and normal tissues. The x−axis "T" (Red) represents cancer tissue sample and "N" (Green) represents normal tissue sample. The y−axis is the log2 of the gene expression in the sample.

Figure S7. GO and KEGG enrichment analysis of the highest score modules obtained from MCODE. The left is the genes enriched in each pathway, and the color is the differential multiple gene expression.

Table S1. The number of genes in each module.

Table S2. Core modules.

Table S3. MCC, Degree, and Closeness analysis results.

Table S4. Five enrichment pathways of the core module .

Figure S1

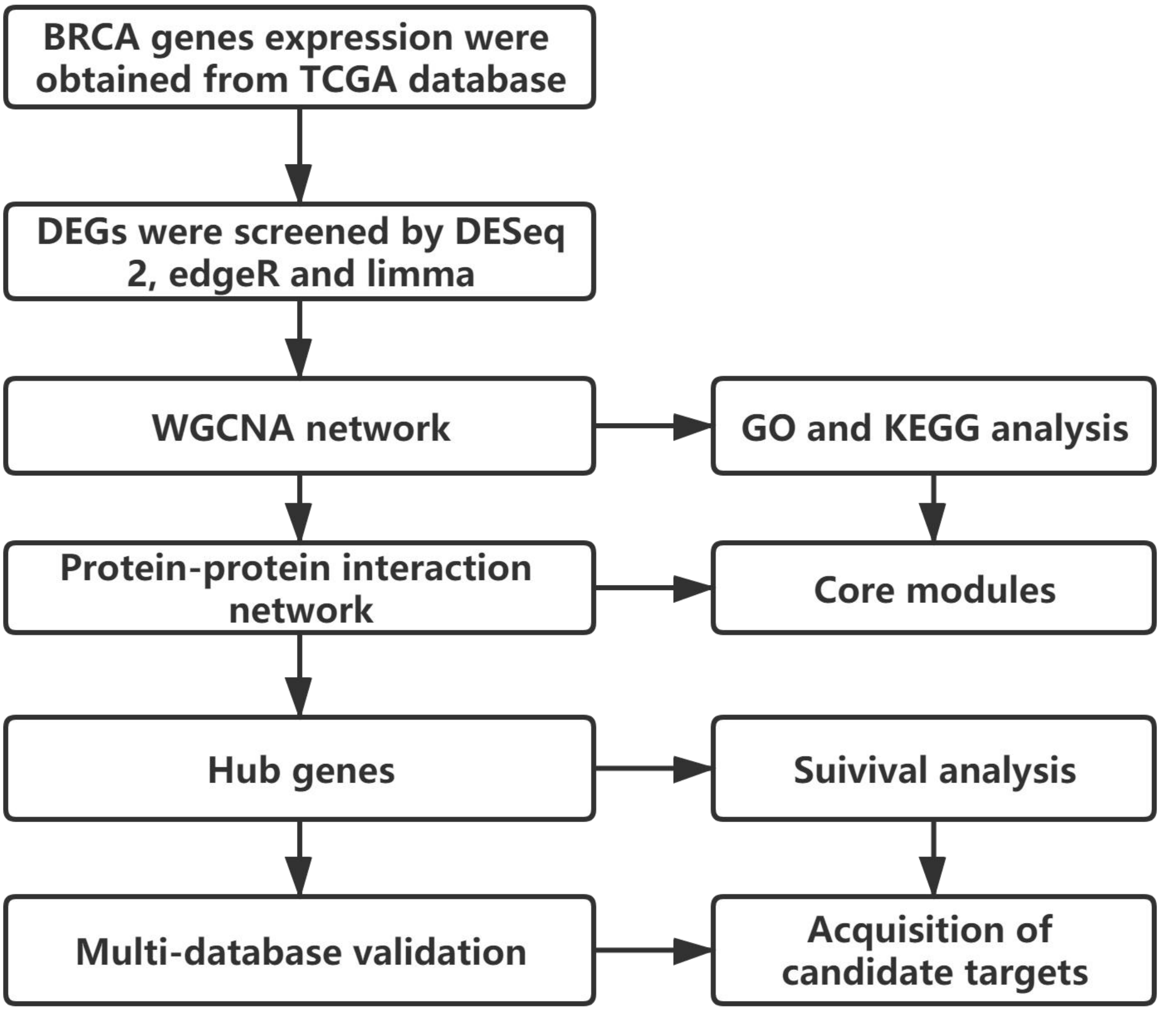
BRCA genes expression were obtained from TCGA database
DEGs were screened by DESeq 2, edgeR and limma
WGCNA network
GO and KEGG analysis
Protein-protein interaction network
Core modules
Hub genes
Suivival analysis
Multi-database validation
Acquisition of candidate targets

Figure S2

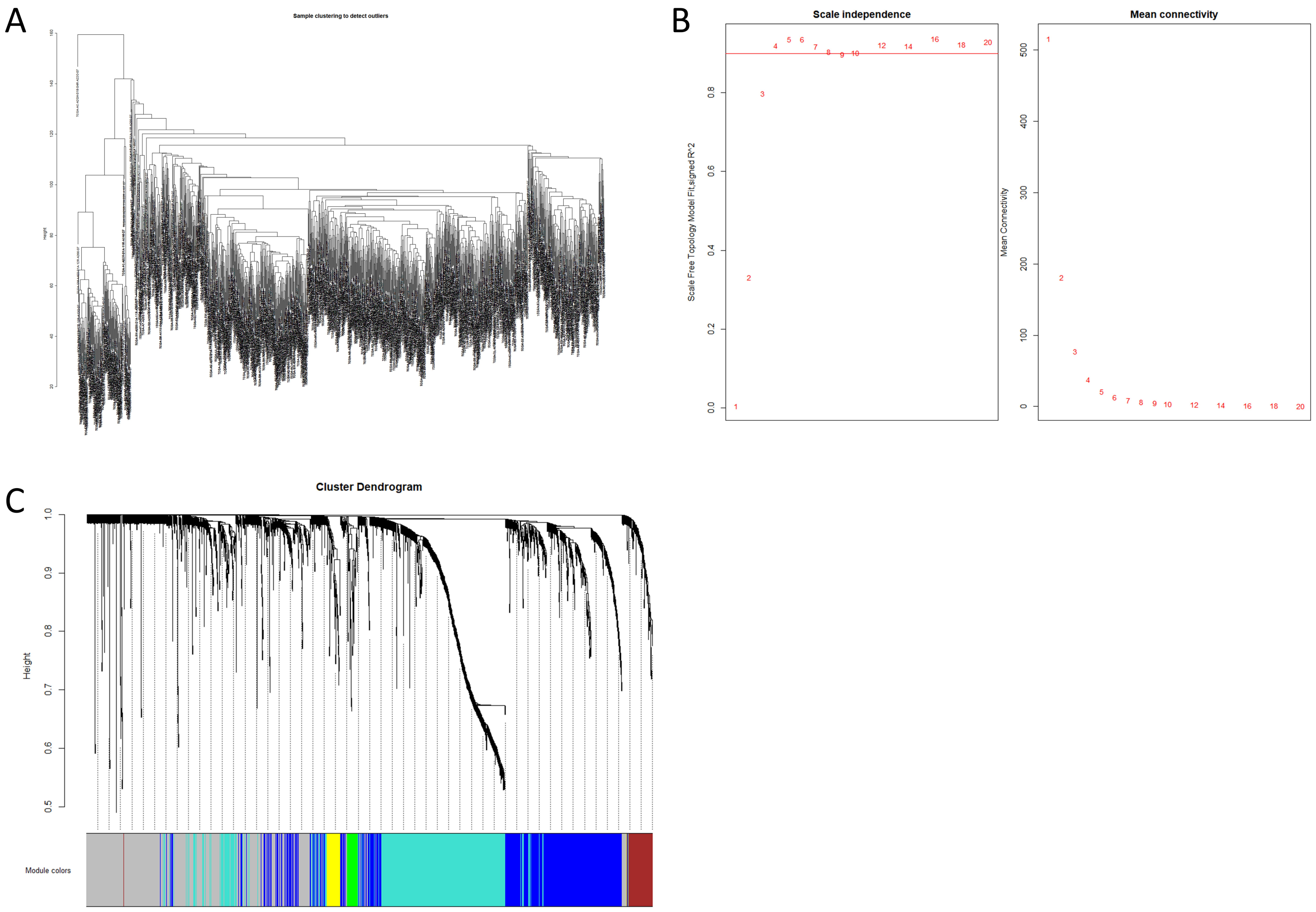

A
Sample clustering to detect outliers
Height
B
Scale independence
Scale Free Topology Model Fit,signed R^2
Mean connectivity
Mean Connectivity
C
Cluster Dendrogram
Height
Module colors

Figure S3

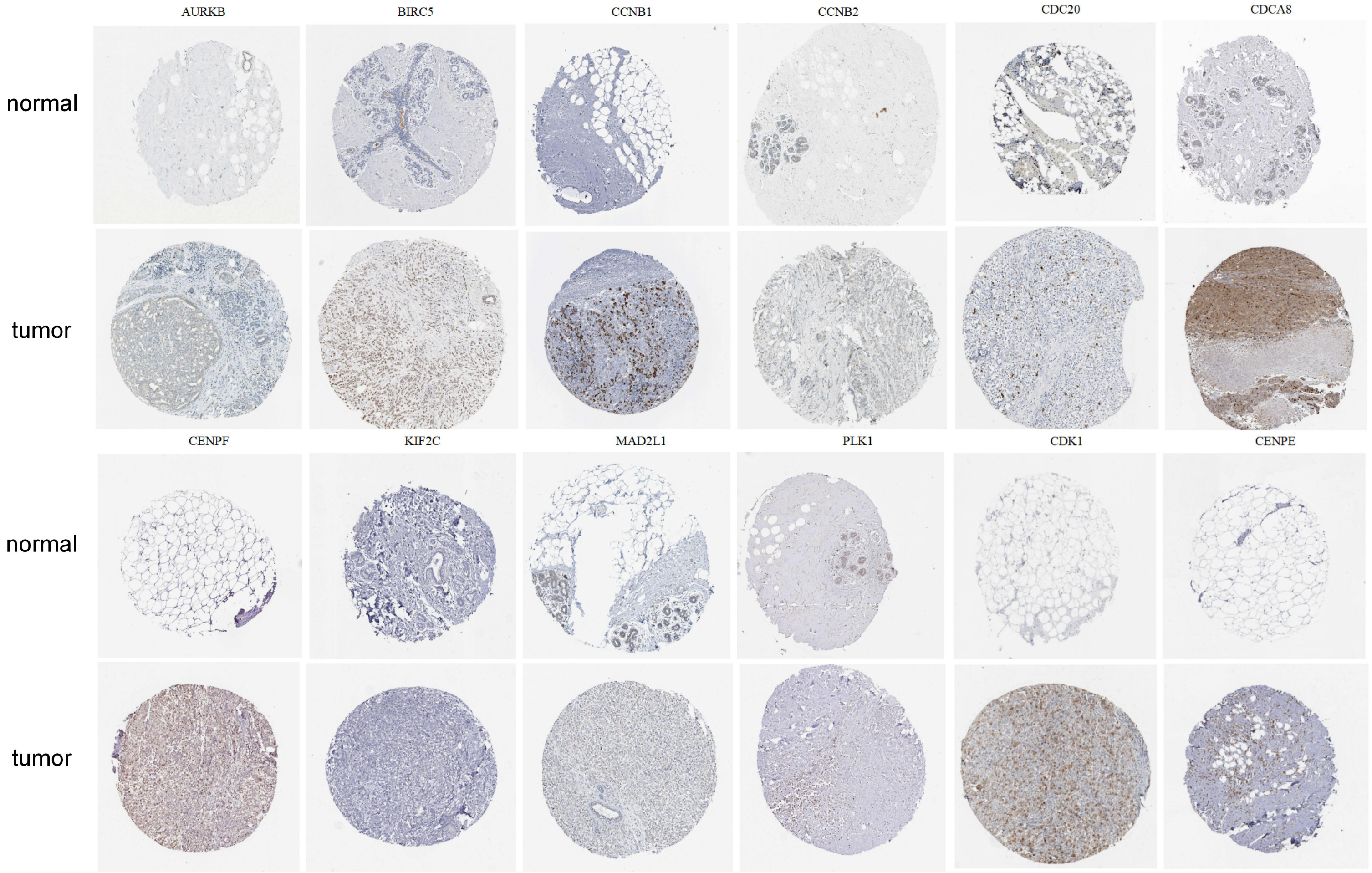
AURKB
BIRC5
CCNB1
CCNB2
CDC20
CDCA8
normal
tumor
CENPF
KIF2C
MAD2L1
PLK1
CDK1
CENPE
normal
tumor

Figure S4

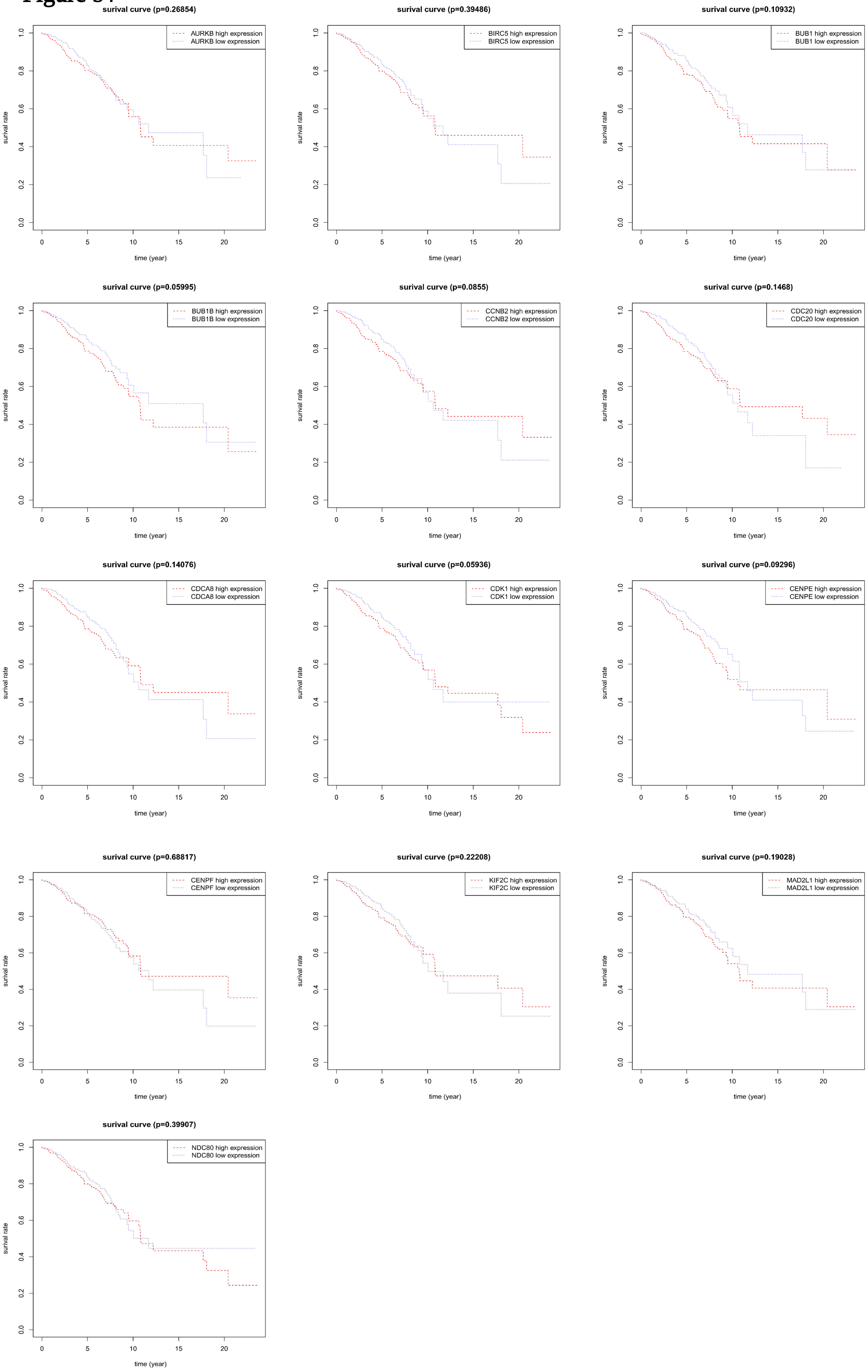

surival curve (p=0.26854)
AURKB high expression
AURKB low expression
surival curve (p=0.39486)
BIRC5 high expression
BIRC5 low expression
surival curve (p=0.10932)
BUB1 high expression
BUB1 low expression
surival curve (p=0.05995)
BUB1B high expression
BUB1B low expression
surival curve (p=0.0855)
CCNB2 high expression
CCNB2 low expression
surival curve (p=0.1468)
CDC20 high expression
CDC20 low expression
surival curve (p=0.14076)
CDCA8 high expression
CDCA8 low expression
surival curve (p=0.05936)
CDK1 high expression
CDK1 low expression
surival curve (p=0.09296)
CENPE high expression
CENPE low expression
surival curve (p=0.68817)
CENPF high expression
CENPF low expression
surival curve (p=0.22208)
KIF2C high expression
KIF2C low expression
surival curve (p=0.19028)
MAD2L1 high expression
MAD2L1 low expression
surival curve (p=0.39907)
NDC80 high expression
NDC80 low expression
surival rate
time (year)

Figure S5

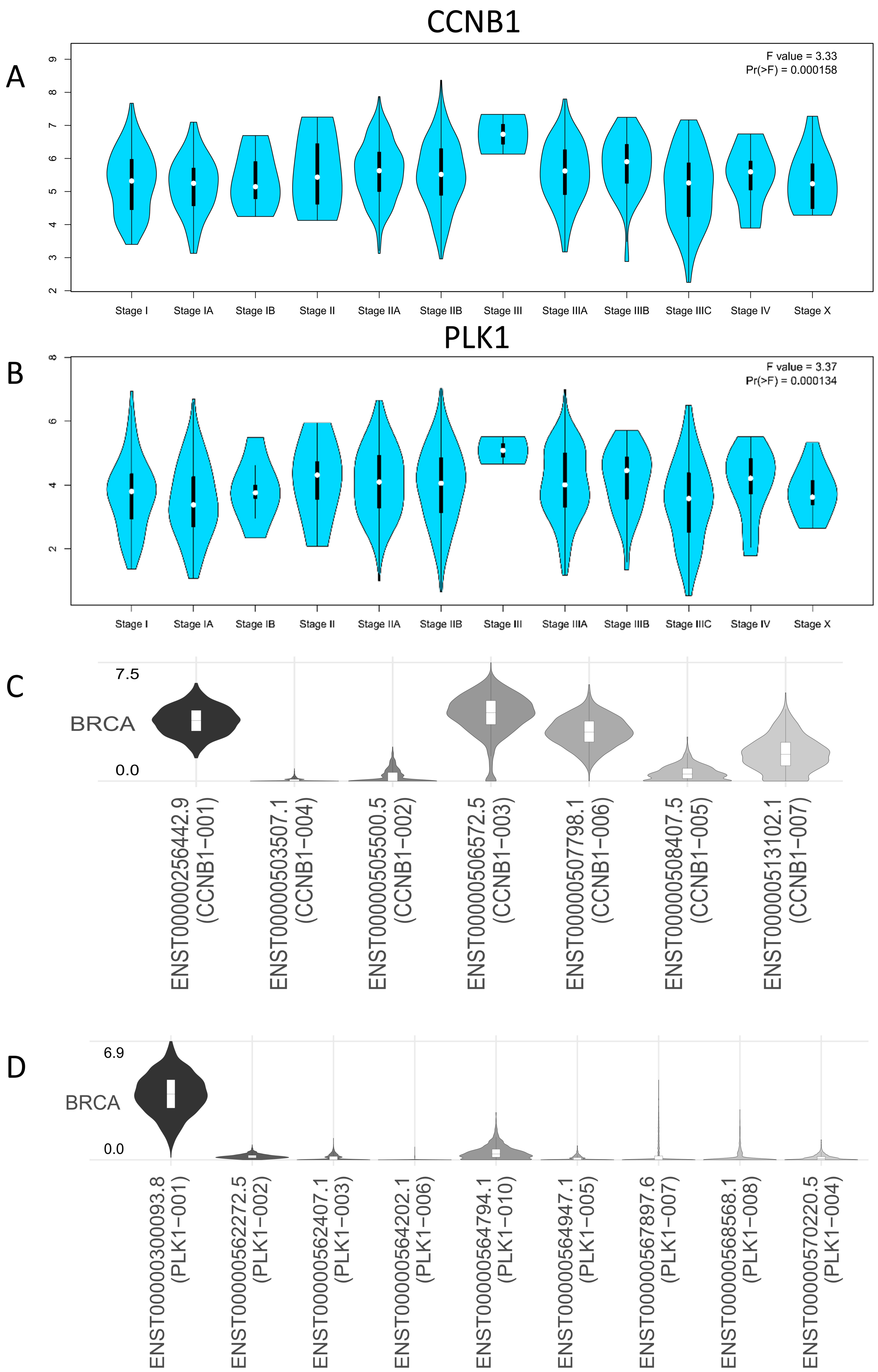
CCNB1
A
F value = 3.33
Pr(>F) = 0.000158
Stage I
Stage IA
Stage IB
Stage II
Stage IIA
Stage IIB
Stage III
Stage IIIA
Stage IIIB
Stage IIIC
Stage IV
Stage X
PLK1
B
F value = 3.37
Pr(>F) = 0.000134
C
7.5
BRCA
0.0
ENST00000256442.9 (CCNB1−001)
ENST00000503507.1 (CCNB1−004)
ENST00000505500.5 (CCNB1−002)
ENST00000506572.5 (CCNB1−003)
ENST00000507798.1 (CCNB1−006)
ENST00000508407.5 (CCNB1−005)
ENST00000513102.1 (CCNB1−007)
D
6.9
BRCA
0.0
ENST00000300093.8 (PLK1−001)
ENST00000562272.5 (PLK1−002)
ENST00000562407.1 (PLK1−003)
ENST00000564202.1 (PLK1−006)
ENST00000564794.1 (PLK1−010)
ENST00000564947.1 (PLK1−005)
ENST00000567897.6 (PLK1−007)
ENST00000568568.1 (PLK1−008)
ENST00000570220.5 (PLK1−004)

Figure S6

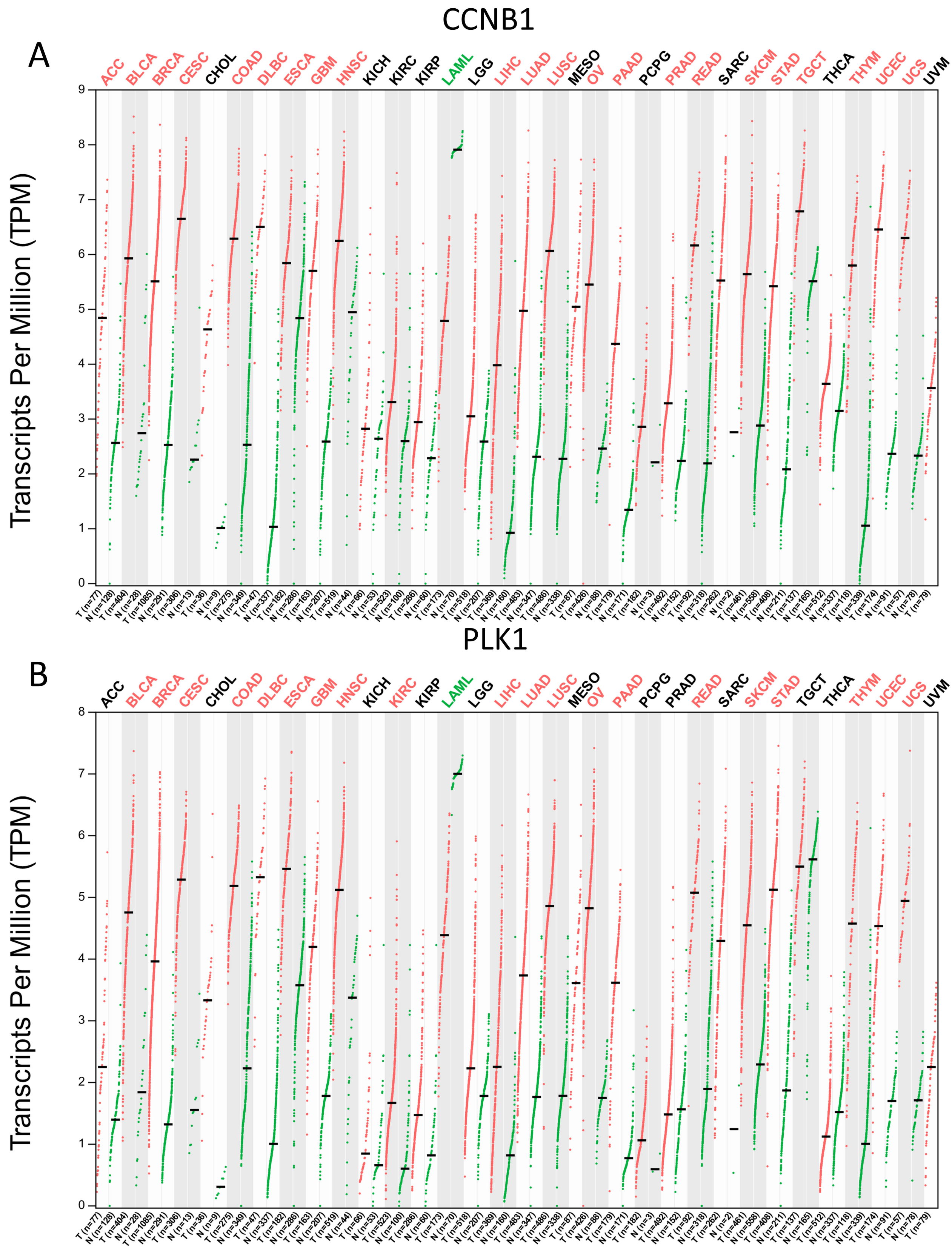
CCNB1
A
ACC BLCA BRCA CESC CHOL COAD DLBC ESCA GBM HNSC KICH KIRC KIRP LAML LGG LIHC LUAD LUSC MESO OV PAAD PCPG PRAD READ SARC SKCM STAD TGCT THCA THYM UCEC UCS UVM
Transcripts Per Million (TPM)
0
1
2
3
4
5
6
7
8
9
PLK1
B
ACC BLCA BRCA CESC CHOL COAD DLBC ESCA GBM HNSC KICH KIRC KIRP LAML LGG LIHC LUAD LUSC MESO OV PAAD PCPG PRAD READ SARC SKCM STAD TGCT THCA THYM UCEC UCS UVM
Transcripts Per Million (TPM)
0
1
2
3
4
5
6
7
8

Figure S7

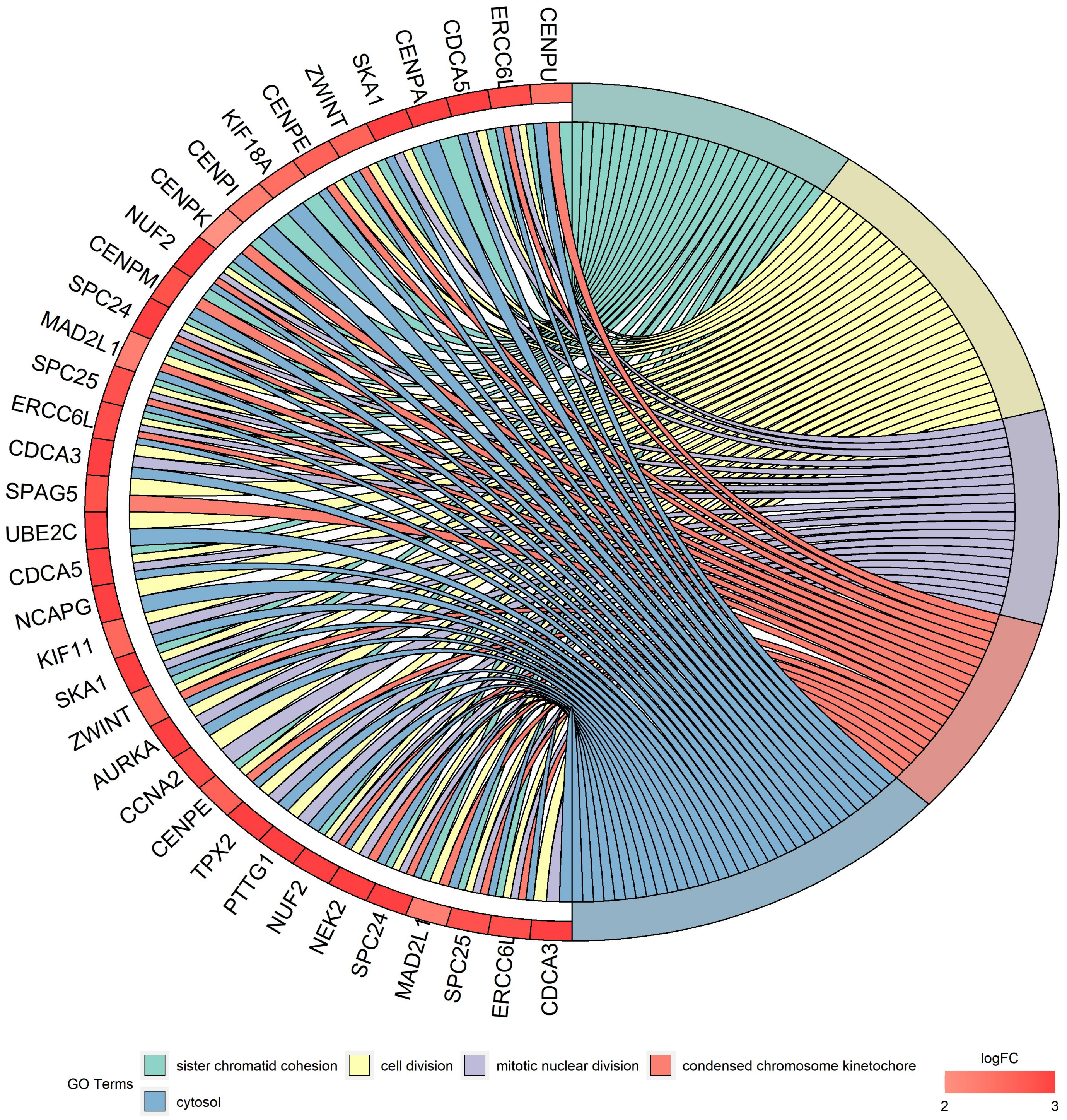

CENPU
ERCC6L
CDCA5
CENPA
SKA1
ZWINT
CENPE
KIF18A
CENPI
CENPK
NUF2
CENPM
SPC24
MAD2L1
SPC25
ERCC6L
CDCA3
SPAG5
UBE2C
CDCA5
NCAPG
KIF11
SKA1
ZWINT
AURKA
CCNA2
CENPE
TPX2
PTTG1
NUF2
NEK2
SPC24
MAD2L1
SPC25
ERCC6L
CDCA3
GO Terms
sister chromatid cohesion
cell division
mitotic nuclear division
condensed chromosome kinetochore
cytosol
logFC
2
3

Table S1. The number of genes in each module

Color of module	Number of genes
blue	594
brown	93
green	38
grey	655
turquoise	600
yellow	52

Table S2. Core modules

Cluster	Score	Nodes	Edges	Node IDs
1	23.561	42	483	CDCA5, CASC5, SGOL1, KIF15, TOP2A, ZWINT, TTK, SPC24, CCNA2, CENPI, CENPM, MELK, ERCC6L, MAD2L1, NUSAP1, KIF20A, CENPK, NUF2, CENPE, CENPA, KIF18A, PTTG1, CEP55, KIF4A, CDCA3, ESPL1, TPX2, PRC1, NCAPG, PBK, KIF11, AURKA, KIF23, ASPM, DLGAP5, RRM2, NEK2, SKA1, SPC25, SPAG5, CENPU, UBE2C
2	16	18	136	CDK1, NDC80, CCNB1, KIF2C, MKI67, BIRC5, BUB1B PLK1, HJURP, KIAA0101, CDCA8, CCNB2, CDC20, RACGAP1, FOXM1, CENPF, AURKB, BUB1
3	10	19	90	ORC6, POLE2, BLM, RAD51, DNA2, EME1, H2AFX, CDC7, BRCA2, XRCC2, MCM2, CDT1, CLSPN, RMI2, ORC1, BRIP1, EXO1, RAD51AP1, MCM10
4	7	7	21	COL5A2, COL5A1, COL11A1, P4HA3, COL22A1, COL10A1, COL1A1
5	7	7	21	NOTUM, VGF, PROC, TMEM132A, CDH2, FN1, MATN3
6	5	5	10	KIFC1, KIF1A, KIFC2, KIF18B, KIF26B
7	5	5	10	RAMP1, PTH1R, SCT, ADM2, ADRB2
8	4.8	6	12	MMP9, MMP13, MMP1, SDC1, MMP3, ACAN
9	4.571	8	16	HTR1D, GRM8, GPR68, NPW, GPR143, NMU, F2RL2, CCL20
10	4.5	5	9	OLR1, STBD1, ADAM8, CLEC5A, PLAUR
11	4	4	6	ZBTB16, UBE2S, CCNF, PARK2
12	4	4	6	ISG15, IRF7, OASL, IFI6
13	3.333	4	5	TYMS, CDC45, MCM4, CDC6
14	3	5	6	E2F1, CDC25C, CCNE1, PKMYT1, CDC25A
15	3	3	3	GINS4, GINS1, GINS2
16	3	3	3	KRT19, KRT18, KRT8

Table S3. MCC, Degree, and Closeness analysis results

MCC	Degree	Closeness
CDK1	CDK1	CDK1
BUB1	CDC20	CDC20
BUB1B	CCNB1	CCNA2
CDC20	CCNA2	CCNB1
CCNB2	CCNB2	CCNB2
CCNB1	BUB1	BUB1
KIF2C	AURKB	PLK1
NDC80	BUB1B	AURKB
CDCA8	PLK1	KIF11
CENPF	CDCA8	BUB1B
BIRC5	KIF11	CDCA8
AURKB	KIF2C	TOP2A
PLK1	TOP2A	KIF2C
MAD2L1	MAD2L1	MAD2L1
CENPE	KIF20A	NDC80
NUF2	NDC80	BIRC5
ESPL1	CENPE	KIF20A
CENPA	BIRC5	ASPM
SGOL1	CENPF	CENPE
KIF18A	ASPM	CENPF

Table S4. Five enrichment pathways of the core module

Category	Term	Count	%	Genes	FDR
GOTERM_BP_DIRECT	GO:0007062~sister chromatid cohesion	15	37.5	CENPU,ERCC6L,CDCA5, CENPA,SKA1,ZWINT,CENPE,KIF18A,CENPI,CENPK,NUF2,CENPM,SPC24, MAD2L1,SPC25	1.01E-19
GOTERM_BP_DIRECT	GO:0051301~cell division	19	47.5	ERCC6L,CDCA3,SPAG5, UBE2C,CDCA5,NCAPG,KIF11,SKA1,ZWINT,AURKA,CCNA2,CENPE,TPX2,PTTG1,NUF2,NEK2,SPC24, MAD2L1,SPC25	1.32E-18
GOTERM_BP_DIRECT	GO:0007067~mitotic nuclear division	17	42.5	ERCC6L,CDCA3,CDCA5, KIF11,SKA1,KIF15,AURKA,CCNA2,ASPM,TPX2,PTTG1,NUF2,PBK,NEK2,SPC24,CEP55,SPC25	5.17E-18
GOTERM_CC_DIRECT	GO:0000777~condensed chromosome kinetochore	12	30	CENPU,CENPE,ERCC6L, SPAG5,NUF2,CENPK,CENPM,NEK2,SPC24,ZWINT,MAD2L1,SPC25	1.32E-15
GOTERM_CC_DIRECT	GO:0005829~cytosol	29	72.5	ERCC6L,CDCA3,CDCA5, NCAPG,KIF11,CENPA,SKA1,AURKA,KIF15,PTTG1,NUF2,NEK2,CENPU,RRM2,UBE2C,KIF23,ZWINT, CENPE,TPX2,KIF18A,ESPL1,PRC1,CENPI,KIF4A, CENPK,CENPM,SPC24,MAD2L1,SPC25	1.02E-11
